# Unlocking longevity: the role of telomeres and its targeting interventions

**DOI:** 10.3389/fragi.2024.1339317

**Published:** 2024-01-25

**Authors:** Marlies Schellnegger, Elisabeth Hofmann, Martina Carnieletto, Lars-Peter Kamolz

**Affiliations:** ^1^ Division of Plastic, Aesthetic and Reconstructive Surgery, Department of Surgery, Medical University of Graz, Graz, Austria; ^2^ COREMED–Centre for Regenerative and Precision Medicine, JOANNEUM RESEARCH Forschungsgesellschaft mbH, Graz, Austria; ^3^ Research Unit for Tissue Regeneration, Repair and Reconstruction, Division of Plastic, Aesthetic and Reconstructive Surgery, Department of Surgery, Medical University of Graz, Graz, Austria

**Keywords:** telomere length, telomerase, longevity, interventions, oxidative stress, inflammation, telomerase activators

## Abstract

Average life expectancy has been steadily increasing in developed countries worldwide. These demographic changes are associated with an ever-growing social and economic strain to healthcare systems as well as society. The aging process typically manifests as a decline in physiological and cognitive functions, accompanied by a rise in chronic diseases. Consequently, strategies that both mitigate age-related diseases and promote healthy aging are urgently needed. Telomere attrition, characterized by the shortening of telomeres with each cell division, paradoxically serves as both a protective mechanism and a contributor to tissue degeneration and age-related ailments. Based on the essential role of telomere biology in aging, research efforts aim to develop approaches designed to counteract telomere attrition, aiming to delay or reduce age-related diseases. In this review, telomere biology and its role in aging and age-related diseases is summarized along with recent approaches to interfere with telomere shortening aiming at well- and healthy-aging as well as longevity. As aging research enters a new era, this review emphasizes telomere-targeting therapeutics, including telomerase activators and tankyrase inhibitors, while also exploring the effects of antioxidative and anti-inflammatory agents, along with indirectly related approaches like statins.

## 1 Introduction

In the past century, the average life expectancy has roughly doubled basically in all developed countries worldwide. Demographic data estimate that by the year 2050, the global population of individuals aged 60 and above is expected to reach 2.1 billion, effectively doubling the figure reported in 2020. Additionally, the number of adults over 80 years old will triple and reach 426 million (United Nations Department of Economic and Social Affairs, 2022). This demographic shift is clearly associated with an escalating social and economic burden, challenging not only for our healthcare system but also the social security systems, workforce dynamics, housing infrastructure, and intergenerational relationships (Partridge et al., 2018). Primarily, the aging process involves a functional decline, which in turn leads to a steady increase in chronic diseases, including cancer, neurodegenerative diseases, diabetes melitus, and cardiovascular diseases (Hou et al., 2019; Bellary et al., 2021; Liberale et al., 2022). These age-associated diseases predictively double in incidence every 5 years after the age of sixty. Furthermore, the WHO designates advanced age as an independent risk factor for major life-threatening disorders. For instance, when considering coronary heart disease, advanced age is rated as an equally significant risk factor as smoking (Melzer et al., 2020).

Therefore, it seems to be of utmost importance to develop strategies and interventions that interfere with the aging process, prevent or at least mitigate age-associated diseases, and support healthy aging patterns, not only to elongate the lifespan but also to increase the healthspan - the phase of life spent in health, active, and in absent of chronic diseases (Seals et al., 2016). Over the past decades, the healthspan has not increased at the same pace as the lifespan and lags far behind; this observed disparity raises significant concerns. The quality of life and wellbeing during aging is to prioritize; otherwise, the challenges posed by an excessively growing aging population will become unmanageable (Gonzalez-Freire et al., 2020). Not surprisingly, the potential to positively intervene and modulate the aging process has been a longstanding area of interest. Specifically, the defined hallmarks of aging are frequently targeted in intervention strategies aiming to mitigate or decelerate the mechanisms of the aging process (Shetty et al., 2018; Campisi et al., 2019). The hallmarks of aging comprise twelve identified variables as crucial contributors to the aging process (López-Otín et al., 2013; López-Otín et al., 2023). These hallmarks encompass genomic instability, the shortening of telomeres, deregulated nutrient sensing, mitochondrial dysfunction, changes in epigenetic regulation, loss of proteostasis, cellular senescence, stem cell exhaustion, and altered intercellular communication (Campisi et al., 2019). Telomere attrition belongs to the cardinal hallmarks of aging and has garnered significant attention in gerontological research over the past years. Telomeres, the protective ends of chromosomes, progressively diminish with each cell division. Once a critical length is reached, cells may undergo senescence or apoptosis, serving as a safeguard against genetic irregularities (Blackburn et al., 2006). While this mechanism has actually protective origins, in the context of aging, it counterintuitively accelerates tissue degradation and ushers in age-related disorders. The central role of telomere biology in aging has led research into therapies designed to counteract telomere attrition, aiming to delay or mitigate age-linked diseases (Aubert and Lansdorp, 2008; Shay and Wright, 2019; Alder and Armanios, 2022). Targeting telomere dynamics presents a promising avenue in gerontology, well-aging, and the development of therapies for age-associated ailments, underlining the importance of understanding telomere dynamics (Blackburn et al., 2015a). Despite telomeres’ established role in aging, the field of telomere biology faces a significant challenge: the lack of effective, clinically proven therapies that directly target telomeres. This gap underscores the complexity of translating fundamental telomere research into therapeutic applications and the challenges in addressing the multifaceted nature of telomere dynamics and their systemic impact on aging and age-related diseases. Therefore, continued exploration and innovative strategies in telomere research are essential to develop tangible, effective treatments for age-related pathologies.

## 2 Telomere biology: the basics and beyond

A comprehensive understanding of the underlying mechanisms of aging and their impact on long-term health provides valuable insights into resilience mechanisms, their influence on stress responses, and the ensuing effects on health (Ferrucci et al., 2020). The defined hallmarks of aging are a fundamental part of current research in aging; each one fulfills three premises: an age-associated manifestation, an experimentally amplification accelerates the aging process, and interventions targeting these hallmarks offer potential to decelerate, maintain, or even reverse aging (López-Otín et al., 2023). Although the individual hallmarks are intricately interwoven, specific ones are selectively targeted to develop intervention strategies and therapeutic approaches.

### 2.1 Telomere shortening: at the nexus of cellular aging

In 2013, telomere attrition was described as a primary hallmark of aging (López-Otín et al., 2013), and its research has increasingly garnered attention in recent years. However, the notion of telomeres emerged already in the 1930s when Creighton and McClintock studied Zea mays and hypothesized the presence of a distinctive structure at chromosome ends, critical for preventing chromosome end fusion (Creighton and McClintock, 1931). Since then, extensive research in this field has been has continually revealed new insights, recognizing its role in the aging process and various diseases (Cawthon et al., 2003; Rizvi et al., 2014; Wang et al., 2018; Yuan et al., 2018).

Telomeres are described as consecutive repeats of the six-nucleotide sequence (TTAGGG) in the form of a cap structure (Blackburn and Gall, 1978), serving to protect chromosome ends from initiating a DNA damage response. Due to the inherent constraints of DNA polymerases in replication, telomeric DNA cannot be completely replicated. This leads to the gradual shortening of telomeres with each cycle of cell division (Allsopp et al., 1992; Rhodes et al., 2002). When telomeres shorten to a critical point, they cause genomic instability, which hinders further replication, leading to senescence and eventually cell death (Blackburn, 2001). At note, DNA polymerase requires a transient primer to commence the unidirectional synthesis from the 5′to the 3′end, but it cannot fully replicate the 3′ ends of the chromosome, which is described as the “end-replication problem” (Levy et al., 1992). Certain mechanisms are required to bypass this end-replication problem, which leads to a milestone in telomere research: the identification of telomerase in 1985 by Greider and Blackburn. Telomerase, a ribonucleoprotein enzyme, elongates chromosomes by adding DNA sequence repeats to their terminal regions, thus facilitating the extension of telomere length (Greider and Blackburn, 1985). Lee et al. showed that in successive generations of mice, late-generation specimens exhibited impaired spermatogenesis and reduced proliferative capacity in bone marrow and spleen. These deficits were associated with significant telomere attrition and chromosomal aberrations, highlighting the critical role of telomerase and telomeres in preserving genomic integrity in high-renewal organ systems (Lee et al., 1998). However, the expression of telomerase is predominantly restricted to stem cells and certain progenitor cells; most somatic cells do not express or only have very low levels of telomerase activity (Martínez and Blasco, 2015). This results in progressive telomere shortening over time, acting as a “biological clock” that caps the number of possible cell divisions (Harley et al., 1990). Although the lack of telomerase in somatic cells is a limiting factor for proliferation, studies emphasize that it is also preventing the uncontrolled growth of the cell, potentially leading to cancer (Akincilar et al., 2016). About 85% of cancer types have been found to reactivate telomerase, allowing cancer cells to maintain their telomeres and thus enabling them to proliferate indefinitely (Shay and Wright, 2005).

In the orchestration of telomere dynamics, the shelterin complex stands out as a crucial player. It is composed of several proteins and binds specifically to telomeric DNA, ensuring telomeres are not erroneously identified as DNA breaks, thereby preventing an inappropriate DNA damage response (de Lange, 2018). Any dysfunction in shelterin components can lead to telomere uncapping, exposing telomeres to degradation, recombination, and chromosomal end-to-end fusions, inducing genomic instability potentially resulting in cellular senescence (Sfeir and de Lange, 2012). TRF1 and TRF2, both essential components of the shelterin complex, help to maintain telomere integrity. Particularly, TRF2 is essential for inhibiting the ATM kinase signaling pathway, a primary responder to DNA double-strand breaks (Karlseder et al., 1999). Further, POT1, another crucial component, attaches to the single-stranded overhang of the telomere, preventing the ATR kinase-mediated DNA damage response. The interplay between telomerase activity, telomere length, and shelterin function is essential for the cellular stability. Imbalances can result in conditions ranging from premature aging to the onset of cancer (Pinzaru et al., 2016). Conversely, this understanding offers also therapeutic targets. For instance, it has been shown that the genetic removal of TRF1 hinders tumor progression in aggressive lung cancer and glioblastoma mice models, which occurred through direct telomere damage, irrespective of telomere length (Bejarano et al., 2017; Bejarano et al., 2019).

### 2.2 Telomeres: telling tales of human aging and disease

Exploring the intricate nexus between telomeres and human aging, a broad body of both *in vitro* and *in vivo* studies, complemented by contemporary human research, has revealed pivotal correlations. These studies demonstrate how changes in telomere length may not only mirror the aging process but also potentially influence the onset and progression of age-related diseases (Zheng et al., 2022). In a Southern Italian cohort of 516 individuals aged 65–106, a notable trend in leukocyte telomere length was observed, with a decline post-70 years and a subsequent increase after 92 years, paralleling demographic survival curves. This trend suggests widespread telomere attrition linked to increased mortality risk and organismal decline, while those in better physical condition exhibited reduced attrition, contributing to delayed senescence (Crocco et al., 2021). Concurrently, a study by Crocco et al. highlighted a minimal genetic impact on leukocyte telomere length in the elderly, challenging the concept of strong genetic control over telomere length in older age groups and underscoring the significant role of chromosomal structure integrity genes, like TERF1 and TNKS2, in longevity (Crocco et al., 2021). Additionally, a meta-analysis of 48,000 individuals identified genetic loci, including TERC and TERT, associated with mean leukocyte telomere length. These loci are implicated in telomere biology and various cancers and age-related diseases, such as idiopathic pulmonary fibrosis. An analysis of these genetic variants revealed that alleles linked to shorter leukocyte telomere length significantly increased coronary artery disease risk, suggesting a causal relationship between telomere-length variation and certain age-related diseases (Codd et al., 2013) Overall, these findings underscore the complex yet critical role of telomeres in the biological mechanisms of aging and disease susceptibility.

### 2.3 Telomere dysfunction in cellular aging: the interaction with inflammation and oxidative stress

In primary human cells, each cell division leads to a reduction of 50–100 bases from the telomeres across all chromosomes. This depletion rate is notably exceeding what the end-replication mechanism would predict, which points to additional factors influencing telomere attrition in human cells (Harley et al., 1990; Blackburn et al., 2015b). Factors including oxidative stress and inflammation are associated with expedited telomere attrition, consequently reducing the replicative lifespan of cells (Kawanishi and Oikawa, 2004; Zhang et al., 2016a). While chronic inflammation is one of the twelve hallmarks of aging identified by López-Otín et al. (2023) oxidative stress, *per se*, is not explicitly listed as a separate hallmark. However, “mitochondrial dysfunction” - which is closely linked to the production of reactive oxygen species (ROS) and oxidative stress (Zhang et al., 2016b; Kausar et al., 2018) - is recognized as one of the twelve key contributors. Undoubtedly, oxidative stress is deeply interwoven with several of the postulated hallmarks and, therefore, worth a closer look.

#### 2.3.1 Oxidative stress and telomers

The “Free Radical Theory of Aging,” introduced in the 1950s by Denham Harman, suggests that the aging process in organisms is due to the cumulative cellular damage caused by free radicals over time. Free radicals, especially reactive oxygen species (ROS), can inflict damage to various cellular macromolecules, with DNA being a prime target (Harman, 1956). This influential approach laid the foundation for subsequent research on aging. Over time, it has been refined and adapted according to emerging insights.

Oxidative stress, emerging from an imbalance between the production of ROS and the cell’s antioxidative defense mechanisms, is significantly detrimental to telomeric regions. Telomeres, with their guanine-rich sequences, are particularly prone to oxidative modifications due to the raised susceptibility of guanine to oxidative damage (Singh et al., 2019). One notable outcome of this vulnerability is the formation of 8-oxo-guanine (8-oxoG) lesions, which is a prevalent DNA damage type caused by ROS. A study by Fouquerel et al. demonstrated that 8-oxoG in the telomeric region has a dual role, either hindering telomerase-mediated elongation when incorporated as 8-oxodGTP or promoting telomerase activity by destabilizing G-quadruplex structures when preexisting in telomere DNA. This dual impact of 8-oxoG on telomere function is a key factor in determining whether a cell will experience telomere-related dysfunction or maintain its genomic stability (Fouquerel et al., 2016).

#### 2.3.2 Chronic inflammation and telomers

Over the past decade, the complex interplay between telomere dynamics and chronic inflammation has gained further attention. Evidence suggests that telomere length is closely tied to chronic inflammatory states. Specifically, elevated levels of pro-inflammatory cytokines, such as IL-6 and TNF-α, seem to trigger accelerated telomere shortening (Blackburn et al., 2015a; Deo et al., 2020).

One proposed mechanism suggests that chronic inflammation directly affects telomerase activity. Elevated cytokine levels might suppress telomerase activity, thereby limiting the enzyme’s ability to counteract telomere shortening and leading to cellular senescence (Liu et al., 2023). This senescence can further enhance inflammation by releasing senescence-associated secretory phenotype (SASP) factors, which induces a feedback loop between inflammation and telomere attrition (Herranz and Gil, 2018). Jurk et al. demonstrated in mice that chronic inflammation, induced by the knockout of the nfkb1 subunit of the NF-κB transcription factor, exacerbates telomere dysfunction and cell senescence through a feedback loop involving NF-κB, COX-2, and ROS, thereby leading to premature aging and reduced tissue regeneration in liver and gut (Jurk et al., 2014). These findings underline the importance of managing chronic inflammation to preserve telomere integrity, potentially delaying the onset of age-related diseases.

#### 2.3.3 Interconnected dynamics of cellular aging: *the telomere-mitochondrial axis*

Telomere shortening can disrupt normal cellular function and is implicated in the increased production of ROS, which further contribute to mitochondrial dysfunction and cell aging (Sahin and DePinho, 2010). On the other hand, mitochondria are the primary producers as well as targets of ROS. An excessive accumulation of ROS can lead to damaged mitochondrial DNA (mtDNA), which in turn may induce further mitochondrial dysfunction. This dysfunction can exacerbate ROS production, creating a detrimental feedback loop that significantly contributes to cell aging and age-related pathologies (Akbari et al., 2019).

One proposed mechanism involves the tumor suppressor protein p53. Dysfunctional telomeres can trigger the activation of p53, which in turn may inhibit the transcription of the peroxisome proliferator-activated receptor gamma coactivator 1-alpha (PGC-1α), which is the primary regulator of mitochondrial biogenesis and function. Reduced PGC-1α levels can lead to mitochondrial dysfunction and increased ROS production (Sahin and DePinho, 2012).

Cells with critically short telomeres can undergo senescence, which is often accompanied by a pro-inflammatory senescence-associated secretory phenotype (SASP). SASP can exacerbate mitochondrial dysfunction and further increase oxidative stress (Wiley et al., 2016; Mohamad Kamal et al., 2020). In short, the telomere-mitochondrial axis captures the dynamic crosstalk between telomeres and mitochondria, both vital to cellular aging. This axis underscores the intertwined effects when either component declines and can be seen a feedback loop between telomere dysfunction and mitochondrial dysfunction.

## 3 Telomere-based interventions: current market landscape for longevity

Telomere dysfunction intensifies the molecular hallmarks of aging, potentially amplifying age-related diseases like neurodegeneration and cancer; conversely, the profound understanding of its underlying mechanisms offers avenues for mitigating aging and its associated disorders (Chakravarti et al., 2021). The maintenance of telomere length, either through genetic interventions or modulating telomerase activity, has been shown to delay cellular aging and extend the healthspan in various model organisms (Bernardes de Jesus and Blasco, 2013). Experimental elongation of telomeres through genetic manipulation or pharmacological means has already shown potential in delaying cellular and tissue aging, suggesting an avenue for therapeutic interventions by targeting the aging process itself (Webb and Zakian, 2016). In the following section, we will elucidate and critically discuss the approaches previously explored to beneficially modulate telomere biology.

### 3.1 Telomere-targeting therapeutics

The telomere complex, crucial for cellular senescence and genomic stability, has become a promising target in age-related research. Recent advances have elucidated potential therapeutic strategies for telomere modulation to address age-related conditions and diseases (Gao and Pickett, 2022; Sagris et al., 2022).

#### 3.1.1 Telomerase activators

Telomerase activation has gained prominence as a potential therapeutic approach for extending telomere length and subsequently, cellular healthspan. As Telomerase catalyzes the addition of TTAGGG nucleotide repeats to chromosome ends, it counteracts telomere attrition resulting of cellular divisions (Greider and Blackburn, 1985; Tsoukalas et al., 2019).

##### 3.1.1.1 TA-65

A prominent agent of telomerase activators is TA-65, a compound derived from the Chinese herb *Astragalus membranaceus*. Studies suggest that TA-65 might activate telomerase, potentially leading to telomere extension (Salvador et al., 2016; Kokubun et al., 2019; Tiendrébéogo et al., 2023). In a randomized, double-blinded, placebo-controlled trial, involving 117 cytomegalovirus-positive adults, supplementation with a low dose (250 U) of the telomerase activator TA-65 led to a significant increase in telomere length over 1 year, while the placebo group experienced a significant reduction in telomere length. A higher dose (1000 U) of TA-65 showed a non-significant trend toward telomere lengthening (Salvador et al., 2016). In another double-blinded, randomized trial with elderly myocardial infarction patients, TA-65 was assessed for its potential in modulating immune cell aging. While TA-65 administration did not affect the proportions of terminally differentiated CD8^+^ T-lymphocytes, it led to significant elevations in major lymphocyte subsets and a notable 62% reduction in high-sensitivity C-reactive protein (hsCRP) levels after 12 months compared to placebo. Additionally, fewer adverse events were observed in the TA-65 group (Bawamia et al., 2023).

##### 3.1.1.2 Cycloastragenol (CAG)

Another postulated telomerase activator is Cycloastragenol (CAG), which is also a compound derived from the Astragalus membranaceus plant. Idrees et al. examined the role of CAG in activating telomerase and its impact on the Klb (β-Klotho) gene in mouse ovaries, a key factor in female fertility and aging. Molecular simulations confirmed CAG’s binding to the hTERT model, and its subsequent application rejuvenated telomerase activity, restoring ovarian health in age-induced and Doxorubicin-induced damage models. These findings highlight CAG’s potential in addressing female infertility via TERT-dependent *β*-Klotho regulation (Idrees et al., 2023).

However, while preliminary findings seem promising, comprehensive clinical trials are essential to ascertain the efficacy and safety in promoting telomere elongation and the associated health benefits.

While these approaches hold promise, they are associated with potential risks. For instance, activating telomerase has been associated with an elevated risk of cancer, given that it may permit cells to proliferate unchecked. As cancer involves the unregulated growth of cells, and telomere shortening serves as a natural limit to cell division, artificially extending telomeres could inadvertently increase the risk of cancer (kumar and Sethi, 2023; Luo et al., 2019). The key lies in striking a balance between improving cellular health by lengthening telomeres and avoiding the promotion of tumorigenesis. Prior to the adoption of these treatments, comprehensive clinical trials and rigorous safety assessments are needed.

#### 3.1.2 Telomerase gene therapy

Telomerase gene therapy is an emerging approach that seeks to address cellular aging by directly modulating telomerase activity in cells. In an *in vivo* study conducted in mice, telomerase gene therapy using an adeno-associated virus to express TERT led to significant health improvements and reduced aging markers without elevating cancer incidence. Remarkably, the treatment extended the median lifespan by 24% in 1-year-old mice and 13% in 2-year-old subjects, underscoring the potential of TERT-focused interventions in aging mitigation (Bernardes de Jesus et al., 2012). Another study in a mouse model investigated the therapeutic potential of telomerase gene therapy using adeno-associated virus 9 (AAV) gene vectors to treat aplastic anemia, which is associated with telomere shortening. AAV9-Tert effectively targeted the bone marrow, lengthened telomeres, and mitigated the symptoms of the disease (Bär et al., 2016). An *in vivo* study investigated the influence of telomere length on health in mice derived from embryonic stem cells with hyper-long telomeres. The mice with hyper-long telomeres exhibited reduced DNA damage with aging, improved metabolic markers such as lower LDL levels, improved glucose and insulin tolerance, decreased cancer incidence, and increased longevity (Muñoz-Lorente et al., 2019). Certainly, direct telomerase gene therapy has not been tested in humans due to safety and ethical concerns, unknown long-term effects, and the technically challenging delivering mechanism. Nevertheless, abandoning the telomerase gene therapy approach may be premature given its potential to revolutionize aging and disease treatment. The challenges in human translation certainly necessitate refined methodologies and advanced clinical trials to bridge the gap, ensuring the approach’s safety and efficacy for human therapeutics.

#### 3.1.3 Tankyrase inhibitors

Tankyrase inhibitors are molecules designed to inhibit the function of tankyrases (Tankyrase 1 and Tankyrase 2), which are enzymes in the poly (ADP-ribose) polymerase (PARP) family (Smith et al., 1998; Smith and de Lange, 2000). A fundamental work in this field was conducted by Huang et al. showing that tankyrases regulate the stability of axin, which is a key component of the *β*-catenin destruction complex. By using a small-molecule inhibitor of tankyrase, XAV939, they showed stabilization of axin and downregulation of Wnt signaling (Huang et al., 2009). The Wnt pathway is indirectly connected to telomere biology through its regulation of adult stem cell function. Essential for stem cell self-renewal and maintenance, Wnt signaling indirectly contributes to telomere length preservation during stem cell divisions. Consequently, any dysregulation in Wnt signaling can affect these regenerative processes, potentially destabilizing telomeres and thereby impacting aging and the onset of age-related diseases (Brack et al., 2007; Liu et al., 2007; Kahn, 2014). By regulating the Wnt pathway, tankyrase inhibitors have garnered interest as potential therapeutic agents, particularly in cancer research, given the crucial role of dysregulated Wnt signaling in tumor progression and metastasis (Waaler et al., 2012; Huang et al., 2020; Neiheisel et al., 2022). Even though tankyrases play a significant role in cellular aging due to their influence on telomere maintenance, focused research in this area remains sparse and should be pursued in future research.

### 3.2 Antioxidants and anti-inflammatory agents

**FIGURE 1:**
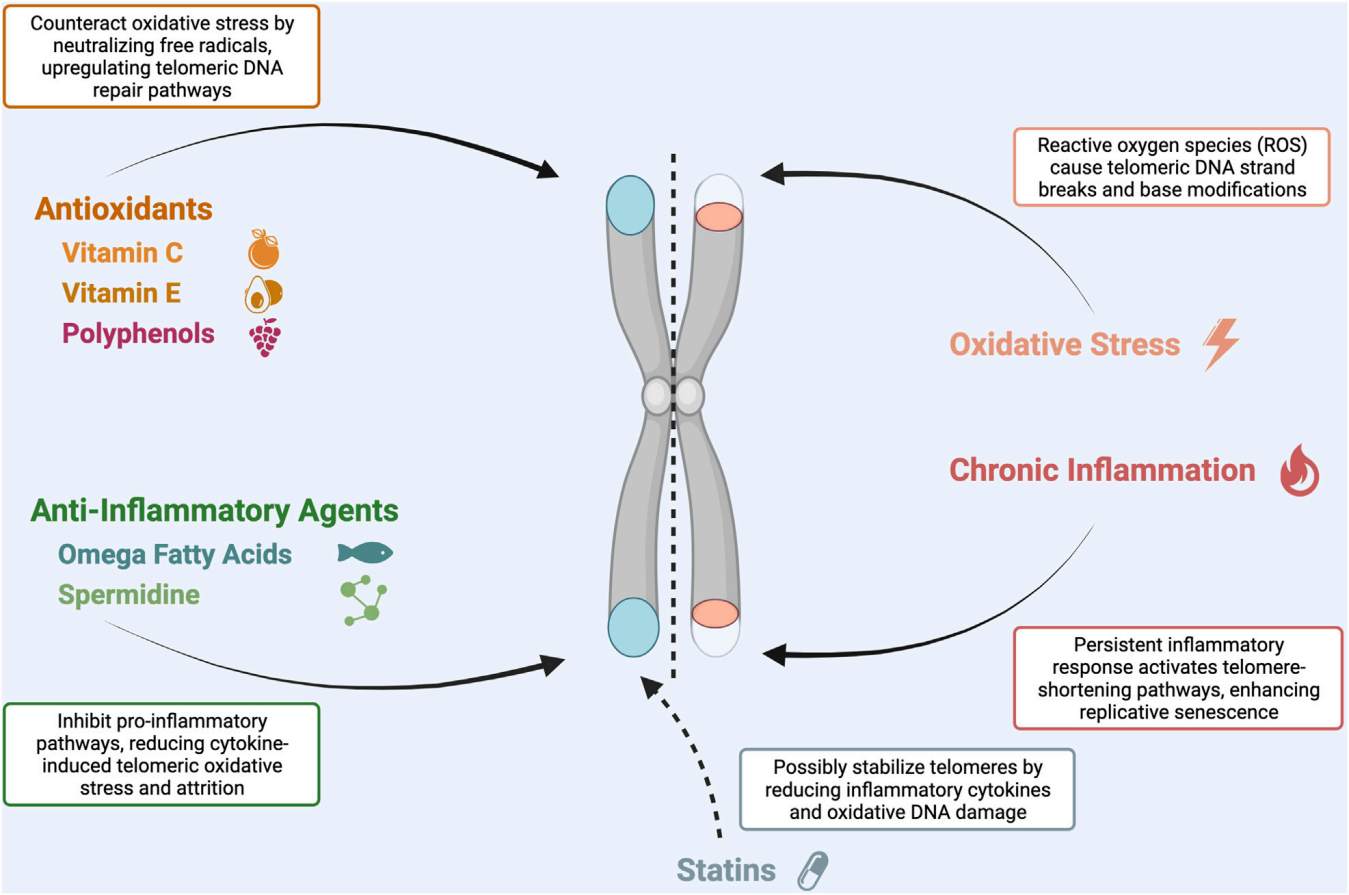
The role of antioxidants and anti-inflammatory agents in terlomere maintenance.

Oxidative stress and chronic inflammation are significant contributors to cellular aging; there is growing evidence linking both to accelerated telomere attrition (López-Otín et al., 2013; Armstrong and Boonekamp, 2023; López-Otín et al., 2023). Consequently, there has been a rising interest in therapies that combat oxidative stress and inflammation to indirectly preserve telomere length, making antioxidants and anti-inflammatory compounds key players in this research field. Antioxidants, such as vitamin C, vitamin E, and polyphenols, neutralize free radicals, potentially mitigating DNA and telomere damage (Crous-Bou et al., 2019). Simultaneously, anti-inflammatory agents, including omega-3 fatty acids and curcumin, could reduce inflammation-mediated telomeric attrition (Siopis and Porter, 2022). An overview of the relevant antioxidants and anti-inflammatory agents influencing telomere maintenance is given in Figure 1.

#### 3.2.1 Antioxidants

Antioxidants neutralize free radicals, which can inflict damage on cellular structures (Halliwell, 1996), including DNA and telomeres. This protective effect can be essential in countering telomere shortening, and thereby possibly delaying cellular aging (Fenech et al., 2023).

##### 3.2.1.1 Vitamin C

Vitamin C, a potent water-soluble antioxidant, scavenges free radicals in the aqueous cellular environment, preventing damage to critical biomolecules. Notably, it can also augment the enzymatic action of telomerase, potentially supporting telomere elongation (Furumoto et al., 1998). Recent studies underscore its capability to enhance telomerase activity, elucidating its integral role in telomere preservation. Among 586 women, the dietary intake of multivitamins and telomere length was observed. Multivitamin users tended to have longer telomeres. Moreover, the micronutrient analysis revealed that especially the vitamins C and E were positively associated with telomere length (Xu et al., 2009). In a recent cross-sectional analysis using the NHANES database with 7.094 participants, a positive correlation between dietary vitamin C intake and telomere length was observed. Specifically, greater vitamin C intake was associated with longer telomeres (Cai et al., 2023). These findings add to the body of evidence that certain vitamins, especially vitamin C, may play a protective role in telomere maintenance, possibly through its antioxidant properties, reducing oxidative stress.

##### 3.2.1.2 Vitamin E

Vitamin E is a lipid-soluble antioxidant, primarily located in cell membranes, with its primary role to protect polyunsaturated fatty acids (PUFAs) from lipid peroxidation, which is a significant source of DNA damage, including telomeres (Corina et al., 2019). In the CORDIOPREV study, involving 1.002 cardiovascular disease patients, dietary intake of vitamin E was found to significantly influence leukocyte telomere length, a biomarker for cellular aging. Patients with inadequate vitamin E intake exhibited shorter telomere length compared to those with sufficient intake (Corina et al., 2019). Another study conducted by Shen et al. examined the connection between DNA telomere length and breast cancer risk, and how antioxidant intake might influence this correlation. Shorter telomeres were associated with a higher breast cancer risk among premenopausal women. While women with the shortest telomeres and low dietary intake of antioxidants, including beta-carotene, vitamin C, and E, showed a moderate increase in breast cancer risk, underlining the importance of an accurate dietary intake of antioxidants (Shen et al., 2009).

##### 3.2.1.3 Polyphenols

Polyphenols, widely present in sources such as fruits, vegetables, or tea, exert antioxidant effects by neutralizing oxidizing species. **Resveratrol**, a polyphenol found highly concentrated in berries, nuts, grapes, and red vine, exerts antioxidant and anti-inflammatory effects (Rotondo et al., 1998; Queiroz et al., 2009; Meng et al., 2021). The anti-inflammatory effect is potentially facilitated through the action of cyclooxygenase, AP1, and NF-κB, although the precise mechanisms remain to be elucidated (Ros and Carrascosa, 2020). The antioxidant effects occur via activating the SIRT1 pathway (Pignet et al., 2021), also a recognized positive regulator of telomere length (Amano et al., 2019; Opstad et al., 2021); it supports cellular defense mechanisms against oxidative and metabolic stress and enhances DNA repair (Palacios et al., 2010; Amano et al., 2019).

Resveratrol has been shown its protective effects on endothelial cells and promoting mitochondrial biogenesis by activating the SIRT1 pathway (Csiszar et al., 2009). An *in vivo* study explored the potential anti-aging effects of a nutraceutical combination of resveratrol and copper in mice. The prolonged administration of resveratrol and copper for 12 months significantly mitigated numerous biological indicators of aging in brain cells, including telomere attrition, amyloid deposition, and DNA damage. Moreover, blood glucose, cholesterol, and C-reactive protein levels were reduced after treatment. These findings suggest that cell-free chromatin particles might contribute to aging processes, and resveratrol and copper could offer therapeutic benefits for promoting healthy aging (Pal et al., 2022).

In human, resveratrol supplementation over 30 days led to metabolic shifts resembling caloric restriction; resveratrol decreased resting metabolic rates, reduced blood glucose, triglycerides, liver enzymes, and inflammation markers, and improved muscle cellular energy mechanisms and insulin sensitivity (Timmers et al., 2011). In a double-blind, placebo-controlled trial with 40 post-infarction patients, resveratrol supplementation significantly enhanced left ventricular diastolic function and endothelial function while reducing LDL-cholesterol levels. Furthermore, resveratrol effectively protected against adverse shifts in red blood cell deformability and platelet aggregation (Magyar et al., 2012).

Given the existing evidence, there is increasing interest in resveratrol as a potential therapeutic agent for promoting telomere maintenance and healthy aging.

#### 3.2.2 Anti-inflammatory agents

Prolonged inflammatory responses can increase oxidative stress and DNA damage, potentially accelerating telomere attrition. Several studies have been investigated anti-inflammatory agents and its influence on telomere biology, being discussed in the following section.

##### 3.2.2.1 Omega-3 fatty acids

Omega-3 fatty acids, particularly eicosapentaenoic acid (EPA) and docosahexaenoic acid (DHA), are essential polyunsaturated fatty acids (PUFAs) recognized for their anti-inflammatory properties. Emerging research indicates a potential role for these fatty acids in telomere biology (Chang et al., 2020; Ogłuszka et al., 2022). A prospective cohort study by Farzaneh-Far et al., examining 608 patients with stable coronary artery disease found that individuals with higher blood levels of EPA and DHA had reduced rates of telomere shortening over a 5-year period, suggesting a protective effect of omega-3 fatty acids on telomeres. The proposed mechanisms underlying these observations include the ability of omega-3s to reduce oxidative stress and systemic inflammation. Moreover, omega-3 fatty acids might modulate the activity of telomerase, and thereby extending telomeric DNA (Farzaneh-Far et al., 2010). O'Callaghan et al. (2014) investigated the potential of omega-3 fatty acid supplementation to attenuate telomere shortening in elderly individuals with mild cognitive impairment. The findings suggest that omega-3s might play a role in telomere maintenance, which could have implications for aging and neurodegenerative diseases. While the study emphasizes the connection between telomere length, cognitive decline, and omega-3 supplementation, it adds to the growing body of evidence on the potential benefits of omega-3s in cellular aging (Barden et al., 2016; Liu et al., 2021). Also, dietary patterns such as a mediterranean diet, known to be rich in omega-3 fatty acids, is associated with longer telomeres, and thereby can be linked to healthier aging (Canudas et al., 2020).

However, even though there is growing evidence highlighting beneficial effects of several antioxidants and anti-inflammatory agents, findings are not consistent. A cross-sectional study examining 263 postmenopausal women, the relationship between leucocyte telomere length and various dietary factors, including vitamins and antioxidants like anthocyanidin. Although the main analysis found no significant association between telomere length and the dietary patterns, an exploratory observation did note a connection between anthocyanidin intake and telomere length, which, after further correction, emerged non-significant (Mickle et al., 2019).

##### 3.2.2.2 Statins

Statins are a class of drugs commonly prescribed to lower cholesterol levels by inhibiting the enzyme HMG-CoA reductase (Endo, 1992), thereby reducing the risk of cardiovascular diseases (Baigent et al., 2010). The relationship between statins and telomere length has been an area of interest, but the evidence is not entirely conclusive. However, there is the assumption that they might influence telomere length through anti-inflammatory and antioxidative effects or by increasing the activity of telomerase, the enzyme responsible for maintaining telomere length. Some observational studies have suggested that statin users tend to have longer telomeres compared to non-users, which could imply a potential protective effect of statins on cellular aging (Brouilette et al., 2003). In a cross-sectional study analyzing 3.496 adults, no significant difference in leukocyte telomere length between statin users and nonusers was observed. A non-statistically significant trend indicated longer telomeres with prolonged statin use, but potential biases could not be out (Tran et al., 2018). Further, individuals with shorter mean leucocyte telomere lengths were found to be at a higher risk of developing coronary heart disease events. The risk was nearly doubled in those with shorter telomeres among placebo-treated patients, but this increased risk was significantly reduced in those treated with pravastatin (Brouilette et al., 2007). Considering these findings, statins likely influence telomere stability indirectly through their systemic anti-inflammatory and antioxidative effects. This mitigating effect on inflammation and oxidative stress implies that statins could potentially slow down telomere attrition, thus contributing to an overarching effect of aging deceleration. However, more profound research, especially high-quality studies are needed to draw definitive conclusions about the influence of statins on telomere biology.

##### 3.2.2.3 Spermidine

Spermidine is a polyamine implicated in cellular autophagy and anti-inflammatory pathways (Gabandé-Rodríguez et al., 2019), has been demonstrated to influence telomere stability and elongation. Further, spermidine is associated with its cardio-protective effects. An *in vivo* study showed that spermidine intake in mice enhances cardiac autophagy, mitophagy, and mitochondrial respiration, and reduces cardiac hypertrophy and systemic inflammation, which are closely linked to age-related cardiovascular disease (Eisenberg et al., 2016). Also, a prospective cohort study emphasized the potential cardioprotective effect, which might be mediated by its influence on oxidative stress markers, although the exact mechanism was not totally comprehended (Yu et al., 2022). Other studies, including those by Eisenberg et al., provide evidence that spermidine supplementation can extend the lifespan of different organisms; the lifespan was significantly prolonged by counteracting oxidative stress, possibly through the epigenetic downregulation of histone H3 acetylation, thereby suppressing oxidative damage and necrosis. This modulation resulted in the transcriptional activation of autophagy-related genes, culminating in enhanced autophagy (Eisenberg et al., 2009). Another study described that a 6-month regimen of spermidine supplementation in aged mice significantly mitigated various age-related physiological deteriorations, notably improving brain glucose metabolism, reducing cardiac inflammation, lowering liver and kidney pathological conditions, and decreasing age-induced hair loss. Further, spermidine supplementation was associated with reduced rates of telomere shortening (Wirth et al., 2021). While the findings underscore spermidine’s potential as an agent for healthy aging, the underlying mechanisms and its effects on telomeres and longevity require further elucidation in human studies.

## 4 Conclusion and future directions

Over the past decades, medicine has already undergone a notably transformation, shifting from a “sick care” approach, which centered mainly on the treatment of diseases after manifestation, to a “healthcare” paradigm that proactively identifies and mitigates specific risk factors to prevent the manifestation of diseases. Nevertheless, considering the demographic shift towards a progressively aging population worldwide, an even more health-focused approach must be pursued to not only prevent a collapse of the healthcare system but also other socioeconomic structures. A deep understanding of the mechanisms and pathological processes involved in aging is crucial not only for refining therapies for age-related diseases but also for positively influencing the aging process, thereby extending the prospect of a longer, active lifespan. In this context, the telomere complex seems to have a pivotal role. Consequently, unraveling the complexities of telomere biology could unlock potential strategies for tackling age-associated diseases and modulating the aging process itself. Although the described therapeutic approaches and interventions targeting telomere dynamics show some promise, further high-quality human studies and detailed investigations are needed to substantiate any recommendations.

## Abbreviations

CAG, Cycloastragenol; mtDNA, mitochondrial DNA; ROS, reactive oxygen species; SASP, senescence-associated secretory phenotype; TERRA, telomeric repeat-containing RNA; TL, telomere length.
